# Grip force makes wrist joint position sense worse

**DOI:** 10.3389/fnhum.2023.1193937

**Published:** 2023-05-31

**Authors:** Lin Li, Shuwang Li

**Affiliations:** Department of Physical Education, Renmin University of China, Beijing, China

**Keywords:** joint position sense, proprioception, wrist, grip force, joint reposition test

## Abstract

**Background:**

The purpose of this study was to investigate how grip force affects wrist joint position sense.

**Methods:**

Twenty-two healthy participants (11 men and 11 women) underwent an ipsilateral wrist joint reposition test at 2 distinct grip forces [0 and 15% of maximal voluntary isometric contraction (MVIC)] and 6 different wrist positions (pronation 24°, supination 24°, radial deviation 16°, ulnar deviation 16°, extension 32°, and flexion 32°).

**Results:**

The findings demonstrated significantly elevated absolute error values at 15% MVIC (3.8 ± 0.3°) than at 0% MVIC grip force [3.1 ± 0.2°, *t*(20) = 2.303, *P* = 0.032].

**Conclusion:**

These findings demonstrated that there was significantly worse proprioceptive accuracy at 15% MVIC than at 0% MVIC grip force. These results may contribute to a better comprehension of the mechanisms underlying wrist joint injuries, the development of preventative measures to lower the risk of injuries, and the best possible design of engineering or rehabilitation devices.

## Introduction

To maintain postural status, joint position, and overall position in space, the body must be able to convey afferent information from its periphery to the central nervous system (CNS) through proprioception (Riemann and Lephart, 2002; Roijezon et al., 2015). Kinesthesia, joint position sense (JPS), and perception of force are the three subcategories of conscious proprioceptive senses (Riemann and Lephart, 2002). JPS is described as having the capacity to precisely replicate a certain joint angle (Winter et al., 2005; Smith et al., 2009). Wrist joint position sense is especially important for manual dexterity (Goble, 2010).

Previous studies on wrist joint position sense were all carried out with the hand unloaded (Kimura et al., 2021; Wiebusch et al., 2021; Kopruluoglu et al., 2022). However, many tasks call for the hand to exert force while also precisely moving the wrist (Forman et al., 2021). Examples include holding a wrench and rotating your wrist to screw in a screw in an industrial environment, holding a tennis racket and moving your wrist to accurately hit a ball during sports, or holding the steering wheel and turning the car in daily life. Frequent and expensive issues that are increasing are cumulative trauma disorders (CTDs) of the wrist (King et al., 2021; Horino et al., 2022). CTDs are thought to be caused by high power exertion, repetitive motion, and uncomfortable wrist and hand postures (Birkbeck and Beer, 1975; Silverstein et al., 1987; Asadi et al., 2020). We currently do not know the magnitudes of these factors or how they interact (Cázares-Manríquez et al., 2021; Short et al., 2022). Because tasks can be planned with more precise positioning in mind to reduce the incidence of CTDs, it is crucial to understand the link between grip force and wrist joint position sense.

Studies have previously examined how deviating wrist postures influence the maximal grip force (Susta and O’Connell, 2009; Burssens et al., 2017; Bhuanantanondh et al., 2018) or grip force sense (Lu et al., 2017; Li et al., 2020b) and found that maximum grip force or grip force sense decreased as the wrist postures diverged from neutral. Other studies have focused on determining how a constant grip force affects wrist joint ROM (Marshall et al., 1999; Dimartino et al., 2004) and found that as grip force increased, wrist joint ROM decreased. Increasing grip force was also considered one determinant of wrist stiffness (Holmes et al., 2015; Weinman et al., 2021; Mannella et al., 2022).

To the best knowledge of the author, no study has been written up that looks at how a consistent grip force influences the perception of the wrist joint’s location. The relationship between wrist joint position sense and grip force must be understood to better understand the causes of joint injury, develop preventative measures to reduce the risk of injury, and construct engineering or rehabilitation solutions that are both effective and efficient.

## Materials and methods

### Participants

A total of 22 healthy individuals participated in the current investigation (11 men and 11 women, all right-handed) with an average age of 19.0 ± 0.3 years, a weight of 57.6 ± 1.9 kg, and a height of 168.4 ± 1.5 cm. The Edinburgh Handedness Inventory was used to gauge the handedness of each participant (Oldfield, 1971). The laterality quotient was ≥60 (laterality quotient: 90.5 ± 2.5). The adults were untrained for the activity and did not exhibit any neuromuscular abnormalities.

### Apparatus

The joint position sense test system (JPSS, HLTX Technologies, Beijing, China) is a motion study system that has been utilized for measuring joint angles. The JPSS is small (38 × 33 × 20 mm), lightweight (30 g), and ambulatory (i.e., it can be employed anywhere and at any time and is not limited to a specific measurement volume in a laboratory). A JPSS connected to the hand was utilized to calculate the joint angles of the wrist given its three-dimensional orientation. A line was drawn from the wrist’s midpoint to the tip of the index finger to ensure proper alignment. The wireless JPSS sensor’s base was placed on the flat dorsum of the wrist, and its lower edge was positioned away from the wrist and along this line. Before conducting experiments, the device was validated, and the manufacturer calibration settings were employed. Some studies performed with JPSS have shown satisfactory reliability (Cutti et al., 2008; Parel et al., 2012; van den Noort et al., 2014; Li et al., 2015, 2019, 2020a; Marini et al., 2017). The JPSS sampling frequency used in this investigation was 50 Hz.

**FIGURE 1:**
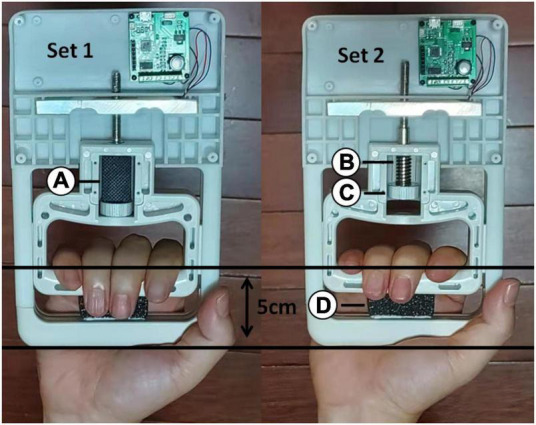
An electronic digital force dynamometer was used for the maximal voluntary isometric contraction test and joint position reposition tests. Inelastic metal support **(A)** was added to the electronic digital force dynamometer during the MVIC test. When the grip distance was 5.0 cm (touching black sponge **(D)**, the grip strength was adjusted to 15% MVIC by turning the knob **(C)** to adjust the spring **(B)**.

With the aid of an electronic digital force dynamometer, strength tests were conducted (grip analyzer; HLTX Technologies, Beijing, China; Figure 1). The instrument was calibrated in accordance with the manufacturer’s recommendations. Prior testing was also carried out to avoid errors. The grip span of the dynamometer was set at 5.0 cm. Inelastic metal support (Figure 1A) was added to the electronic digital force dynamometer during the MVIC test, and 0% MVIC grip force was added during the wrist joint position reposition tests (Figure 1 Set 1). It was removed when the grip force was maintained at 15% MVIC during the wrist joint position sense test (Figure 1 Set 2). When the grip distance was 5.0 cm (touching black sponge, Figure 1D), the grip strength was adjusted to 15% MVIC by turning the knob (Figure 1C) to adjust the spring (Figure 1B). The sampling frequency for this study was set at 100 Hz.

### Protocol

**FIGURE 2:**
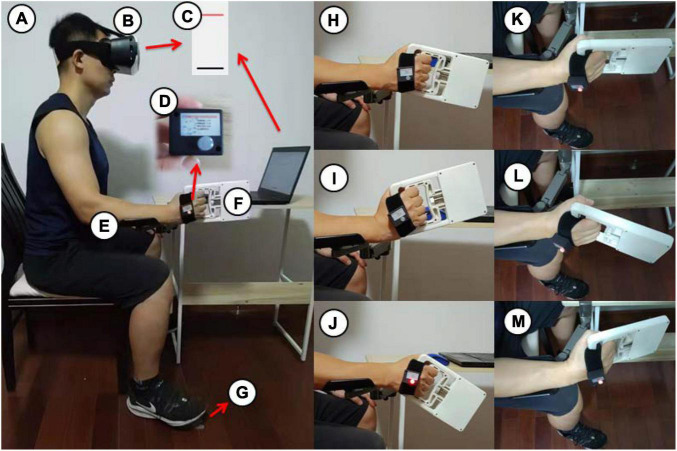
The wrist joint reposition measurements were taken using a consistent body position **(A)** with a head mounted display **(B)**, a screen displayed in the head mounted display **(C)**, JPSS **(D)**, a brace **(E)**, an electronic digital force dynamometer **(F)** and a footswitch **(G)**. The wrist start position **(H)** for radial deviation **(I)** and ulnar deviation **(J)**, the start position **(K)** for extension **(L)** and flexion **(M)**.

To guarantee optimal concentration, this exam was conducted in a quiet room. The participants had a head-mounted display fitted (V-8, Beimu Technologies, Shenzhen, China; Figure 2B). The participant saw the target angle (red line) and the real-time wrist joint angle (black line; Figure 2C), while the display restricted visual feedback from hand motion.

**FIGURE 3:**
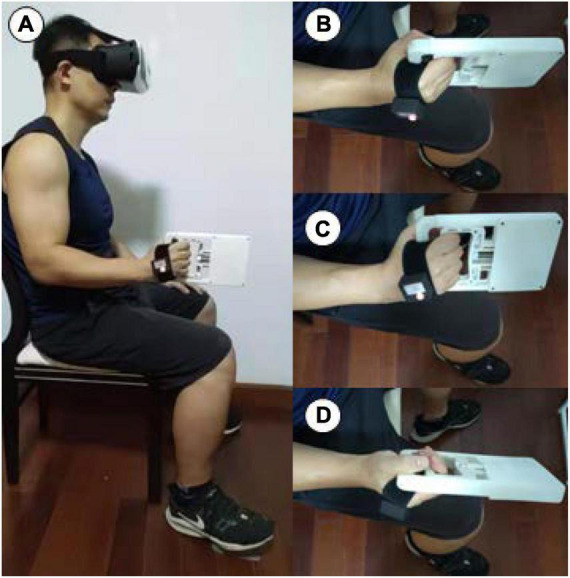
The individuals sat in a chair and the forearm unsupported **(A)**, and the wrist in start position **(B)** for pronation **(C)** and supination **(D)**.

The individuals sat in a chair and then adopted a full body position in a line, with the upper arm adjacent to the body, the forearm placed on a horizontal platform (Figure 2A) or unsupported (Figure 3A), and the wrist in neutral positions (Figures 2H, K, 3B). To guarantee consistency in wrist placement between trials, the forearm firmly rested on the brace (Figure 2E; van den Noort et al., 2014) during wrist extension (Figure 2L), flexion (Figure 2M), radial deviation (Figure 2I), and ulnar deviation (Figure 2J) but was unsupported during pronation (Figure 3C) and supination (Figure 3D). The JPSS was fastened to the participant’s hand using a cushioned, nonelastic strap (Figure 2D). The data were collected and analyzed utilizing a computer, a modified MVIC test, and a joint position sense test program (HLTX Technologies, Beijing, China).

### Maximal voluntary isometric contraction test

Prior to testing, the participants engaged in a warm-up activity. The warm-up exercise included three repetitions of reaching the dynamometer’s submaximal grip force (Jansen et al., 2008). They were instructed to utilize a hand grip and apply the strongest possible grip pressure on the dynamometer. The maximum result from the second test was reported as the grip strength (Bao and Silverstein, 2005). In addition, the participants were given 2 min to relax in between tests to reduce the effect of fatigue on the research results.

### Joint position sense test

**FIGURE 4:**
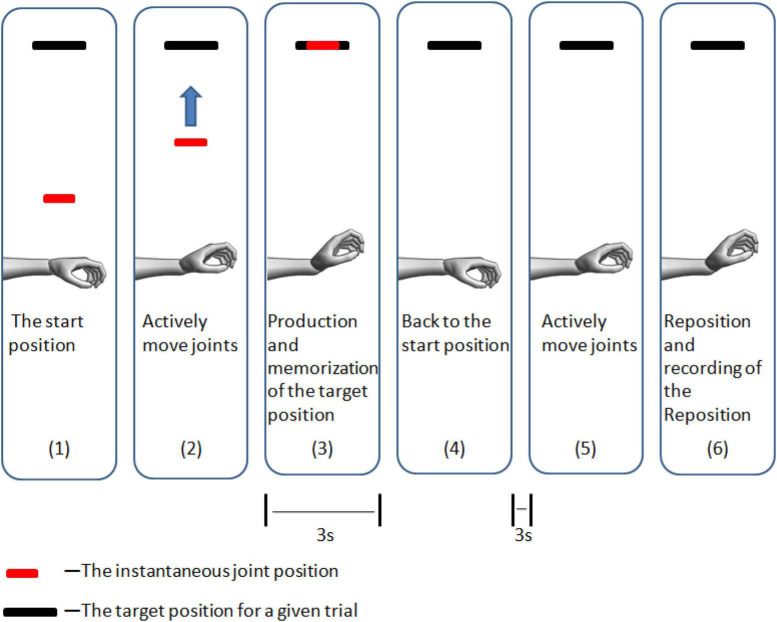
Schema for the computer output observed during the monitoring that instructs the participants to find the target position (wrist extension as an example).

The computer screen was displayed in the head mounted display synchronously. The testing protocol was completely illustrated to participants when they watched the visible output via the head mounted display. One screen with a black line and a red line was shown to participants through the C++ software environment. The black and red lines indicate the wrist joint position and target position, respectively, for a certain trial. All tests started with the joint in the start position (Figures 2H, K, 3B). Participants moved their wrist joint until the black line was inside the red line, showing that the wrist joint had achieved the target position T. Participants were told to hold the target wrist position for 3 s, merely focusing on the wrist joint position. After the participants were kept in the target position for 3 s, the red line disappeared, and a voice programmed into the computer software instructed the participants to rest and move their joint to the start position. The participants tried to regain the target position using no visualization after 3 s. The participants pressed the footswitch with their right foot after establishing that the wrist joint was in the desired position (Figure 2G), and the computer recorded the reproduced position as Ri (*i* = 1,2,3) in the i-trial (Figure 4). The test was conducted thrice. This process was explained to the participants with a demonstration, and they conveniently sensed the process. Practice trials were repeated to enhance the participants’ confidence in performing the task successfully. The target positions (pronation 24°, supination 24°, radial deviation 16°, ulnar deviation 16°, extension 32°, and flexion 32°) and grip force (0 and 15% MVIC) were randomly assigned. The participants held an electronic digital force dynamometer (Figure 2F) in their hands without any extra force when the grip force was 0% MVIC. Low force levels (15% MVIC) were chosen to minimize muscle fatigue (Marini et al., 2017; Naderi et al., 2020). Additionally, some reports states that most everyday manual activities are performed in low force levels (Kotani et al., 2000). To maintain attention on the task during the experiment, the participants rested for 3 min after 9 trials.

### Statistical analyses

Three methods were used to evaluate the position reproduction errors: (1) absolute error (AE), which corresponds to overall error; (2) constant error (CE), which corresponds to error directionality (under- or overestimation); and (3) variable error (VE; for precision), which corresponds to variation in error levels across trials. The following formulas were used to calculate these values:


(1) $$AE=\frac{\sum_{i=1}^{3}\left|R_{i}-T\right|}{3},\left(i=1,2,3\right),$$

(2) $$CE=\frac{\sum_{i=1}^{3}\left(R_{i}-T\right)}{3},\left(i=1,2,3\right),$$

(3) $$VE=\sqrt{\frac{\sum_{i=1}^{3}{\left(R_{i}-\bar{R}\right)}^{2}}{3}},\left(i=1,2,3\right)$$

where the force reproduced during the *i*th trial is denoted by Ri, the target force is denoted by T, the maximum voluntary isometric contraction is denoted by MVIC, and the mean force reproduced over the course of three trials is denoted by.

The sex differences in MVIC values were compared using the independent *t*-test. Mixed-model ANOVAs were utilized as well to examine the effects of position (pronation 24°, supination 24°, radial deviation 16°, ulnar deviation 16°, extension 32°, and flexion 32°), force level (0 and 15% MVIC), and sex (men and women) on AE and VE; in this model, sex was regarded as a between-participants component, while position and force level were considered within-participants variables. Significant effects were rechecked based on extra comparisons, and the *post hoc* least significant difference (LSD) test was applied for multiple comparisons. Additionally, one-sample *t*-tests were performed to compare constant error values for each direction and force level to 0 to identify the trials in which participants produced excessive or insufficient movement. The statistical analysis was carried out using SPSS 22.0 (IBM, Armonk, NY, USA). The level of statistical significance was set at a *P*-value < 0.05, and all results are shown as the mean and standard deviation ( ± SD).

## Results

### Maximal voluntary isometric contraction

The findings demonstrated considerably elevated grip forces in men (359.4 ± 23.1 N) compared to women [247.0 ± 16.4 N, *t*(20) = 3.964, *P* < 0.001]. The 15% MVIC of men (53.9 ± 3.5 N) was also higher than that of women [37.0 ± 2.5 N, *t*(20) = 4.016, *P* < 0.001].

### Absolute error

**FIGURE 5:**
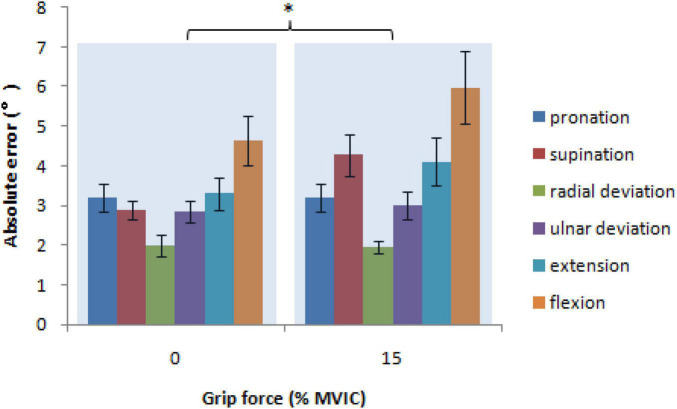
The absolute error, a measurement of the overall error in position reproduction as a function of grip force and wrist position (* = *P* < 0.05).

Mixed-model ANOVA was employed to compute the absolute error, demonstrating no significant interaction among sex, wrist position, grip force, *F*(5,100) = 1.056, *P* = 0.389, sex and grip force, *F*(1,20) = 0.038, *P* = 0.848, sex and wrist position, *F*(5,100) = 0.709, *P* = 0.618, or wrist position and grip force, *F*(5,100) = 1.549, *P* = 0.182. For absolute error, the wrist position [*F*(5,100) = 10.358, *P* < 0.001] and grip force [*F*(1,20) = 5.303, *P* = 0.032] had highly significant effects, but sex did not [*F*(1,20) = 1.018, *P* = 0.325]. There were significantly higher absolute error values at 15% MVIC (3.8 ± 0.3°) than at 0% MVIC grip force [3.1 ± 0.2°, *t*(20) = 2.303, *P* = 0.032; Figure 5]. There were significantly higher absolute error values at flexion (5.3 ± 0.7°) than at pronation [3.2 ± 0.3°, *t*(20) = 2.902, *P* = 0.009], supination [3.6 ± 0.3°, *t*(20) = 2.727, *P* = 0.013], radial deviation [2.0 ± 0.2°, *t*(20) = 5.431, *P* < 0.001], ulnar deviation [2.9 ± 0.2°, *t*(20) = 3.990, *P* < 0.001], and extension [3.7 ± 0.4°, *t*(20) = 3.110, *P* = 0.006]. There were significantly lower absolute error values at radial deviation than those at pronation [*t*(20) = −3.404, *P* = 0.003], supination [*t*(20) = −4.834, *P* < 0.001], ulnar deviation [*t*(20) = −3.617, *P* = 0.002], extension [*t*(20) = −5.256, *P* < 0.001], and flexion [*t*(20) = −5.431, *P* < 0.001].

### Variable error

**FIGURE 6:**
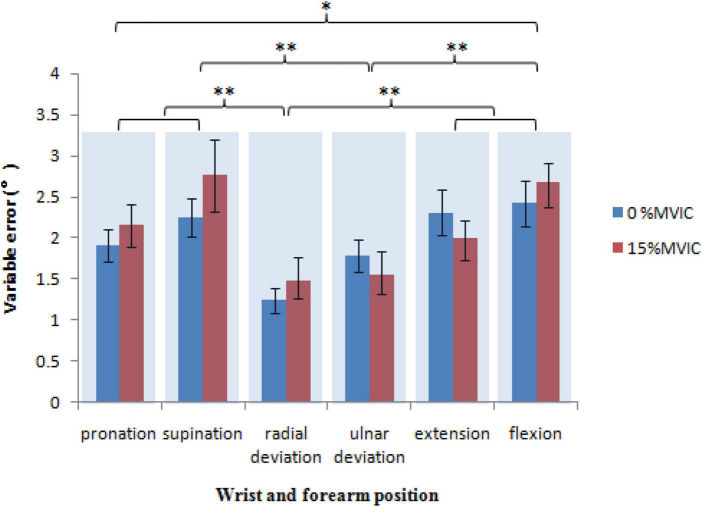
The is the variability in error over several trials. It demonstrates how precise the perform variable error ance is as a function of the grip force and wrist position (* = *P* < 0.05, ** = *P* < 0.01).

Mixed-model ANOVA was used to compute the variable error, revealing no significant interaction between sex, wrist position, and grip force, *F*(5,100) = 0.548, *P* = 0.740, sex and grip force, *F*(1,20) = 0.027, *P* = 0.870, sex and wrist position, *F*(5,100) = 0.348, *P* = 0.882, or wrist position and grip force, *F*(5,100) = 0.729, *P* = 0.604. For variable error, the wrist position [*F*(5,100) = 6.446, *P* < 0.001] had highly significant effects, but not sex [*F*(1,20) = 0.014, *P* = 0.906], and grip force [*F*(1,20) = 0.526, *P* = 0.477]. There were significantly lower variable error values at radial deviation (1.4 ± 0.1°) than those at pronation [2.0 ± 0.2°, *t*(20) = −3.035, *P* = 0.007], supination [2.5 ± 0.3°, *t*(20) = −4.317, *P* < 0.001], extension [2.2 ± 0.2°, *t*(20) = −3.454, *P* = 0.003], and flexion [2.5 ± 0.2°, *t*(20) = −5.988, *P* < 0.001]. There were significantly lower variable error values at ulnar deviation (1.7 ± 0.2°) than those at supination [*t*(20) = −2.935, *P* = 0.008] and flexion [*t*(20) = −3.932, *P* < 0.001]. There were significantly higher variable error values at flexion than those at pronation [*t*(20) = 2.163, *P* = 0.043; Figure 6].

### Constant error

**FIGURE 7:**
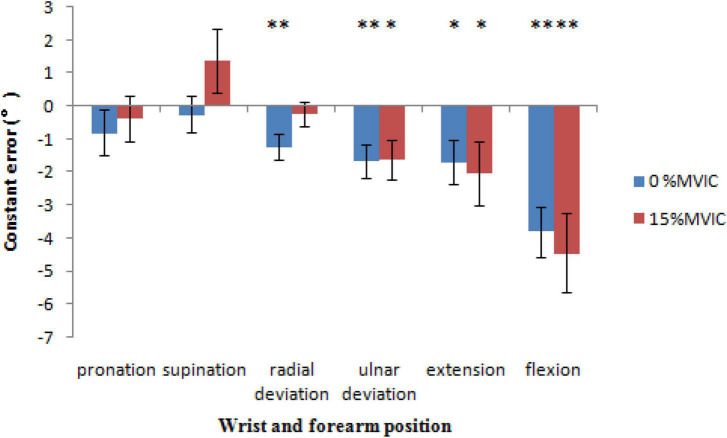
The constant error, reproduction force directionality of error as a function of sex, grip force and wrist position (* = *P* < 0.05, ** = *P* < 0.01).

Significantly lower constant error values were detected at grip force at 0% MVIC of grip force in radial deviation [−1.2 ± 0.4°, *t*(21) = −3.124, *P* = 0.005], ulnar deviation [−1.7 ± 0.5°, *t*(21) = −3.211, *P* = 0.004], extension [−1.7 ± 0.7°, *t*(21) = −2.524, *P* = 0.020], and flexion [−3.8 ± 0.8°, *t*(21) = −4.987, *P* < 0.001] and 15% MVIC of grip force in ulnar deviation [−1.6 ± 0.6°, *t*(21) = −2.702, *P* = 0.013], extension [−2.0 ± 1.0°, *t*(21) = −2.100, *P* = 0.048], and flexion [−4.4 ± 1.2°, *t*(21) = −3.750, *P* = 0.001]. Moreover, estimations were most accurate for wrist joint position at 0 [−0.8 ± 0.7°, *t*(21) = −1.157, *P* = 0.260] and 15% MVIC [−0.4 ± 0.7°, *t*(21) = −0.547, *P* = 0.590] of grip force in pronation, 0 [−0.3 ± 0.6°, *t*(21) = −0.469, *P* = 0.644], and 15% MVIC [1.4 ± 0.9°, *t*(21) = 1.450, *P* = 0.162] of grip force in supination, and 15% MVIC [−0.2 ± 0.4°, *t*(21) = −0.651, *P* = 0.522] of grip force in radial deviation (Figure 7).

## Discussion

Our data were consistent with our previous (Li et al., 2019) and other studies (Marini et al., 2016, 2017; Albanese et al., 2021) showing that the wrist position sense is anisotropic across the joint. Our data also showed significantly worse proprioceptive accuracy (higher absolute error values) at 15% MVIC than at 0% MVIC grip force but not precision (variable error) or directionality (constant error).

Proprioception is a sense that is influenced by the CNS, nerve, and receptors. Because the wrist joint position sense was studied with and without grip load, neurological factors such as the size of the motor cortex (Penfield, 1950; Kocak et al., 2009), the distance and speed of nerve conduction, and the number and distribution of receptors did not change. Grip force may not directly affect neurological factors.

Joint position was sensed through joint, skin, and muscle receptors (Proske and Gandevia, 2012). At normal ranges of joint movement, muscle receptors seem to be the primary source of proprioceptive signals (Proske et al., 2000). Ferrell and Smith claim that skin and joint receptors provide proprioceptive information mostly near the outside limits of a joint’s natural range of motion, presumably acting as limit detectors (Clark et al., 1985; Ferrell and Smith, 1988). The wrist joint deviation range was moderate in this study, so muscle receptors are the major source of proprioceptive signals (Verschueren et al., 1998). The monotonic association of the maintained spindle firing rate with muscle length is utilized in the CNS to obtain information concerning muscle length and limb position (Kokkorogiannis, 2004). Our results revealed no significant difference in the precision (variable error) and directionality (constant error) of wrist joint position sense error between 0 and 15% MVIC grip force. Variable error is the divergence from the mean positioning and is most likely brought on by sensor noise, which is the uncertainty of the sensors (Arnold and Docherty, 2006). Consequently, the muscle spindle discharge rate did not vary throughout the execution of the test no significant difference in the precision (variable error) of wrist joint position sense error between 0 and 15% MVIC grip force. Our results also show that subjects undershot the target position and obtained a smaller constant error at most wrist joint positions. The reason for the smaller ranges when reproducing position may be due to the role of proprioception as a protective mechanism. Subjects subconsciously produce smaller ranges as an overstretch protective mechanism to guard against mechanical and metabolic damage (King et al., 2013; Li et al., 2020a). The constant error was respective to the target position (Goble, 2010; Rinderknecht et al., 2016; Li et al., 2019). Therefore, grip force may not affect constant error.

Grip force shortens the length of the grip muscles (Huxley, 1973; Petrofsky et al., 1980; Lee et al., 2009). According to Hall and McCloskey (1983) proportionate alterations in muscle fiber lengths determine proprioception (Refshauge et al., 1995, 1998; Sturnieks et al., 2007). All of the limb joints have similar detection thresholds when measured in terms of the proportional change in the average muscle fascicle lengths for the muscles crossing them, implying that the brain is interested in fascicle length and shortening as signaled by muscle spindles when predicting joint movement (Refshauge et al., 1998). Our results showed a significantly higher absolute error at 15% than at 0% MVIC grip force. When the same wrist joint angle changes, grip muscles with a shorter length at 15% MVIC grip force have less proportional changes in muscle fiber lengths in the case of the same motion range as the muscles at 0% MVIC grip force, resulting in greater absolute errors.

The flexor digitorum profundus (FDP) and flexor digitorum superficialis (FDS) exert pressure on the median nerve, which is situated underneath the transverse carpal ligament and subject to mechanical pressures (Ugbolue et al., 2005; van Doesburg et al., 2012). Earlier studies detailed the deformation of the median nerve under the hand grip using ultrasound imaging (van Doesburg et al., 2010; Yoshii et al., 2013). A hand grasp causes the FDS tendon to shift palmarly toward the transverse carpal ligament, putting the median nerve under contact stress (Yoshii et al., 2009). Additionally, in comparison to a grip without load, the cross-sectional area of the median nerve may be smaller (van Doesburg et al., 2012; Loh et al., 2016) because of the increased finger flexor tendon load and intrusion of the lumbrical muscles into the carpal tunnel (Cobb et al., 1994, 1995; Keir et al., 1997; Cartwright et al., 2014). Ultimately, hand grip impairs wrist position sense by blocking nerve conduction.

**FIGURE 8:**
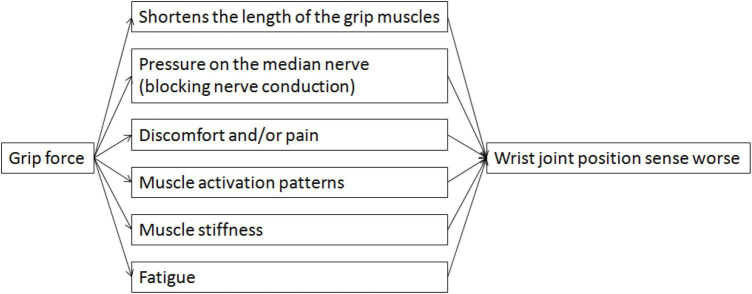
A schematic illustration of the functional link between grip force and wrist joint position sense.

Studies on the effect of grip force on the wrist joint have also shown that grip force involves several peripheral changes, including muscle activation patterns, discomfort, pain, muscle stiffness, fatigue, viscoelastic characteristics (Falzarano et al., 2021), ROM (Marshall et al., 1999; Dimartino et al., 2004), etc. The finger flexor tendons press against the intrawrist structures and the carpal tunnel walls when people perform grip-strength tasks with their wrists in twisted positions, which results in traction, discomfort, and/or pain (self-reported) (Tichauer, 1966; Smith et al., 1977). Similarly, one study found that the majority of participants felt pain when undertaking grip-strength exercises with their wrists in an extreme posture (Pryce, 1980). Pain, which draws attention away from proprioception, may contribute to some of the apparent loss in proprioception (Juul-Kristensen et al., 2008; Anderson and Wee, 2011; Naderi et al., 2020). Proprioception may become compromised in the presence of pain as a result of altered reflex activity and increased sensitivity of the gamma-muscle spindle system (Johansson et al., 2003) brought on by the activation of chemosensitive type III and IV afferents (nociceptors) (Djupsjobacka et al., 1995; Thunberg et al., 2001; Weerakkody et al., 2008; Malmstrom et al., 2013). Moreover, body perception at the central level can be influenced by pain (Rossi et al., 2003; Haggard et al., 2013), including reorganization of the somatosensory cortex (Moseley and Flor, 2012). As a result, pain can impair proprioception both in the peripheral and central nervous systems. Another possible source of the reposition errors considered here was the increased muscle stiffness (Holmes et al., 2015; Weinman et al., 2021; Mannella et al., 2022). Increased muscle stiffness during grip-strength tasks may change proprioceptive receptor responses, such as the responses of the muscle spindles, which would impact the wrist joint position sense (Gregory et al., 1988; Bower et al., 2006; Holmes et al., 2011; Naderi et al., 2020). Another peripheral factor that may reduce proprioception during grip force is peripheral fatigue. Research on how peripheral exhaustion affects proprioception has revealed that fatigue entails a variety of peripheral alterations, such as a changed metabolic state, different muscle activation patterns, and muscular spindle discharge (Enoka and Stuart, 1992; Gandevia, 2001). The high concentrations of metabolites and inflammatory products produced during muscular contraction (for example, lactic acid, arachidonic acid, bradykinin, potassium, and prostaglandin E2) cause an increase in the muscle spindle discharge rate, greater alpha-gamma coactivation, and nociceptor activation in fatigued muscle (Pedersen et al., 1998; Boucher et al., 2012). Therefore, when performing grip-strength tasks, there are significantly higher absolute errors due to the muscle spindle discharge being affected by muscle fatigue. Indeed, several peripheral mechanisms may act simultaneously (Figure 8).

### Limitations

Our study has some limitations. For example, only healthy adult subjects with a mean age of 19.0 years were enrolled in this study. Therefore, the conclusions in the study may only be valid for the assessment of the wrist joint position sense in similarly aged and healthy adult. Thus, additional studies are needed to examine these relationships among other demographics. In addition, there are only two grip force (0 and 15%MVIC) in this study. To check if the relationship between the grip force and wrist position sense likely to be linear or if are there non-linearities, other grip forces (e.g., 30 or 50% MVIC) influence wrist position are needed. Our study has limitations and such grip forces (i.e., 30 or 50% MVIC) are needed in future studies.

## Conclusion

Our study showed a significantly worse proprioceptive accuracy (higher absolute error values) at 15% MVIC than at 0% MVIC grip force, but not precision (variable error) and directionality (constant error). These results may contribute to a better comprehension of the mechanisms underlying wrist joint injuries, the development of preventative measures to lower the risk of injuries, and the best possible design of engineering or rehabilitation.

## Data availability statement

The datasets presented in this study can be found in online repositories. This data can be found here: https://osf.io/gpfmv/.

## Ethics statement

The studies involving human participants were reviewed and approved by the every participant signed informed consent. This work gained approval from the Ethics Review Board of Renmin University of China (reference number 20223231). The patients/participants provided their written informed consent to participate in this study. Written informed consent was obtained from the individual(s) for the publication of any potentially identifiable images or data included in this article.

## Author contributions

LL: conceptualization, data curation, software, writing—original draft, and writing—review and editing. SL: conceptualization, methodology, funding acquisition, supervision, and writing—review and editing. Both authors contributed to the article and approved the submitted version.
